# COVID-19 epidemic curve in Brazil: a sum of multiple epidemics, whose inequality and population density in the states are correlated with growth rate and daily acceleration. An ecological study

**DOI:** 10.1590/0037-8682-0118-2021

**Published:** 2022-02-25

**Authors:** Airandes de Sousa Pinto, Carlos Alberto Rodrigues, Carlito Lopes Nascimento, Lívia Almeida da Cruz, Edval Gomes dos Santos, Paulo Cesar Nunes, Matheus Gomes Reis Costa, Manoel Otávio da Costa Rocha

**Affiliations:** 1 Universidade Estadual de Feira de Santana, Departamento de Saúde, Feira de Santana, BA, Brasil.; 2 Universidade Estadual de Feira de Santana, Departamento de Ciências Exatas, Feira de Santana, BA, Brasil.; 3 Grupo Baiano de Oncologia, Feira de Santana, BA, Brasil.; 4 Universidade Federal de Minas Gerais, Faculdade de Medicina. Programa de Pós-Graduação em Infectologia e Medicina Tropical, Belo Horizonte, MG, Brasil.

**Keywords:** COVID-19, SARS-CoV-2, Polynomial interpolation, Growth rate, Acceleration, Epidemic curve

## Abstract

**Background:**

The epidemic curve has been obtained based on the 7-day moving average of the events. Although it facilitates the visualization of discrete variables, it does not allow the calculation of the absolute variation rate. Recently, we demonstrated that the polynomial interpolation method can be used to accurately calculate the daily acceleration of cases and deaths due to COVID-19. This study aimed to measure the diversity of epidemic curves and understand the importance of socioeconomic variables in the acceleration, peak cases, and deaths due to COVID-19 in Brazilian states.

**Methods:**

Epidemiological data for COVID-19 from federative units in Brazil were obtained from the Ministry of Health’s website from February 25 to July 11, 2020. Socioeconomic data were obtained from the Instituto Brasileiro de Geografia e Estatística (https://www.ibge.gov.br/). Using the polynomial interpolation methods, daily cases, deaths and acceleration were calculated. Moreover, the correlation coefficient between the epidemic curve data and socioeconomic data was determined.

**Results:**

The combination of daily data and case acceleration determined that Brazilian states were in different stages of the epidemic. Maximum case acceleration, peak of cases, maximum death acceleration, and peak of deaths were associated with the Gini index of the gross domestic product of Brazilian states and population density but did not correlate with the per capita gross domestic product of Brazilian states.

**Conclusions:**

Brazilian states showed heterogeneous data curves. Population density and socioeconomic inequality were correlated with a more rapid exponential growth in new cases and deaths.

## INTRODUCTION

Brazil registered the first case of COVID-19 on February 26, 2020, in São Paulo^1^. International air travel was important in the introduction of the initial cases^2^. The country developed one of the highest growth rates for new cases of COVID-19 worldwide^3^, reaching 1,839,850 cases and 71,469 deaths on July 11. However, the epidemic curve does not exhibit homogeneous behavior in the population, and a regional and ethnic variation in mortality due to COVID-19 in Brazil has been described^4^.

The epidemic curve has been obtained, routinely, by the 7-day moving average of the events. Although it facilitates the visualization of discrete variables, which present a large daily variation, it does not allow the calculation of the absolute daily variation rate of new cases per day, that is, the daily acceleration. Recently, we demonstrated that the polynomial interpolation method can be used to accurately calculate the daily acceleration of cases and deaths due to COVID-19^5^. A recent recommendation for the incorporation of this method in the routine analysis of the COVID-19 epidemiological curve was proposed^6^. This method, which applies a methodology that uses the derivative of differential calculus, is used to study curves in several areas of knowledge^7^ ^,^ ^8^.

The measurement of acceleration contributes to the classification of the epidemic curve, comparison of curves between countries and states, in addition to assisting in the application of non-pharmacological measures to address the COVID-19 epidemic. This variable is responsible for the slope of the curve in the ascending and descending phases of the spread, and its lower absolute value in the first phase may indicate the adoption, by countries and states, of more appropriate measures to combat the pandemic, corresponding to a flattened curve in the graph.

Brazil has a high level of socioeconomic inequality that can impact the intensity of the acceleration and peak of daily cases and deaths. This study aimed to measure the diversity of epidemic curves and understand the importance of socioeconomic variables in the acceleration, peak cases, and deaths due to COVID-19 in Brazilian states.

### METHODS

### Study design

This is an ecological study with a time series analysis of new cases and deaths due to COVID-19 in Brazil and its federation units.

### Study design and population

No patients were involved in this study, as secondary data were used. A total of 27 federation units were examined: Acre, Alagoas, Amapá, Amazonas, Bahia, Ceará, Espírito Santo, Goiás, Maranhão, Mato Grosso, Mato Grosso do Sul, Minas Gerais, Pará, Paraíba, Paraná, Pernambuco, Piauí, Rio de Janeiro, Rio Grande do Sul, Rio Grande do Norte, Rondônia, Roraima, Santa Catarina, São Paulo, Sergipe, Tocantins, and Distrito Federal (the Federal District).

### Data source

The time series for each federation unit was obtained from the Brazilian Ministry of Health website (https://covid.saude.gov.br) from February 25 to July 11 (0-137 days of the historical series)^9^. The gross domestic product (GDP), Gini index (GI) of the GDP, and population density (PD) were obtained from the Instituto Brasileiro de Geografia e Estatística (https://www.ibge.gov.br/). The per capita GDP of each state was calculated as the ratio between the GDP and its population. The most recent 2018 GDP data, as well as the estimated population of the states and the GI of the GDP, were utilized^10^ ^-^ ^12^.

The incidence rate was calculated as the ratio between the number of cases accumulated on the last day of the series and the population multiplied by 1,000. Similarly, mortality was obtained by dividing the number of deaths accumulated at the end of the historical series and the population multiplied by 1,000.

### Mathematical modeling

**FIGURE 1:**
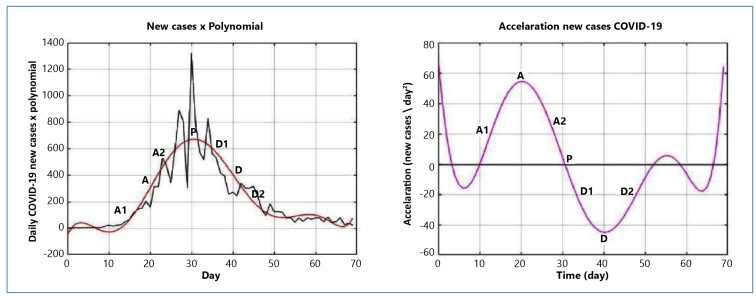
The top figure shows the daily cases of COVID-19 from Austria × polynomial. The bottom figure shows the daily acceleration of COVID-19 from Austria.

The epidemic curve was obtained using the polynomial method in a similar way as in our previous work^5^. The polynomial was automatically generated using the MATLAB software (MathWorks, Matlab R2008a, Natick, Massachusetts, USA) whose degree and coefficients were adjusted to the curve of daily cases and deaths (Figure 1) using the degree at most 8, according to the following equation: $a_{n}{\text{. day}}^{n}+a_{n-1}\cdot {\text{day}}^{n-1}+\ldots .+a_{1}\cdot \text{day}+a_{0^{'}}n\in N$ .

A: maximum acceleration; A1: first stage of the ascending phase, acceleration increases to the maximum value; A2: acceleration remains positive, but the value decreases to zero; P: peak daily cases obtained by the polynomial; D: absolute maximum value of the descending phase of new cases; D1: the first stage of the descending phase, absolute values increase to the maximum value; D2: the second stage of the decreasing curve, deceleration values are reduced to zero.

To illustrate the applicability of the polynomial in describing the epidemic curve, we present data on new cases from Austria (Figure 1), a country that presented a complete curve at the end of the historical series, which did not occur with the curve of any Brazilian state.

Instantaneous acceleration was obtained using the first derivative of the polynomial. The day of the maximum acceleration value and the day of the maximum deceleration value, when present, were determined using the zeros of the polynomial max. In the descending phase of the curve, the negative values of the acceleration were analyzed considering their absolute values and reported as deceleration (Figure 1). In states where the daily acceleration continued to increase until the end of the series, instantaneous acceleration was evaluated up to 5 days before day 137, the end date of the series, to avoid polynomial instability, which was observed at the end of the acceleration curve.

The peak days of new infections were evaluated using the zeros of the first derivative of the polynomial. The epidemic curve of each state was classified according to the values of the cases and acceleration. The COVID-19 infection graph was used to classify the epidemic into phases: ascending, peak, and descending. The acceleration graph enabled subdivision of these phases. The ascending phase presents the first stage with a concomitant increase in new infections and acceleration and the second stage in which numbers continue to increase, but acceleration gradually decreases. At the peak, the number of new cases stabilizes, and the acceleration reaches zero. The descending phase also has two stages. In the first stage, a decrease in new cases and an increase in the absolute value of the deceleration are observed. In the second stage, the event value to decrease, but the acceleration returns to zero (Figure 1).

### Ethical approval

Ethical assessment was not required.

### Statistical analysis

The correlations of socioeconomic data, incidence rate, mortality, maximum acceleration, peak of cases, and deaths were measured by the Pearson correlation coefficient if they presented normal or normalized distribution after logarithmic transformation. The Spearman method was used to assess the correlation between the variables with non-normal distributions.

Normality analysis was performed using the Kolmogorov-Smirnov and Shapiro-Wilk tests. The distribution was considered normal if both tests were met simultaneously. P-values < 0.05 were considered statistically significant.

## RESULTS

### Cases

**FIGURE 2:**
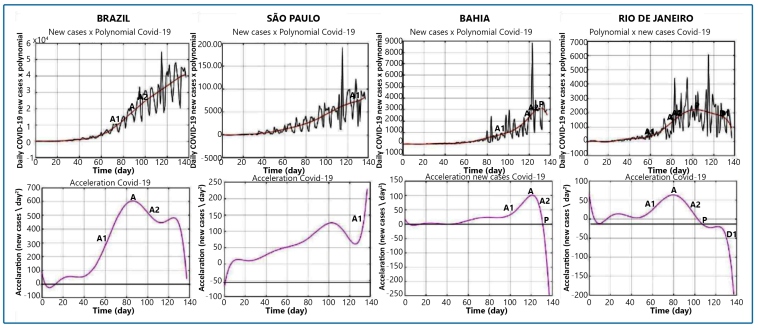
The top figures show the new cases of COVID-19 in Brazil, São Paulo, Bahia, and Rio de Janeiro × polynomial. The bottom figures show the daily acceleration of new cases of COVID-19 in Brazil, São Paulo, Bahia, and Rio de Janeiro. A: maximum daily acceleration; A1: first stage of the growth phase;

A2: second stage of the growth phase; P: acceleration equal to zero (peak); D1: first stage of the descending phase.

On July 11, 2020, the last day of the historical series, Brazil reached 1,839,850 cases, with an incidence rate of 8.94 cases/1,000 inhabitants. Brazil presented a maximum acceleration of 604.7 cases/day^2^ on May 21st, day 86 of the series. The most recent acceleration, day 132, is equal to 373.75 new cases/day^2^, showing a reduction, but still remaining high. As it presented a decrease in acceleration, the country was in the second stage of the acceleration phase (Figure 2). The values by state are listed in Table 1.

**TABLE 1:** Socioeconomic variables and variables obtained in the Covid-19 epidemic curves of Brazilian states.

States	PER CAPITA GDP	PD (N/km^2^)	GI	Total cases (n)	Total deaths (n)	IR (new cases/population *1000)	MR (new deaths/population *1000)	MCA (cases/day^2^ )	PC (cases/day)	MDA (deaths / day^2^)	PD (deaths/day)	DD (deaths/ day^2^)
AC	17,636.74	5.45	0.6982	16,080	419	17.98	0.47	9.2	283	0.2	8	
AL	16,375.55	120.37	0.7194	44,633	1,264	13.32	0.38	27.6	946	0.6	21	
AP	20,247.28	6.05	0.7657	31,279	473	36.30	0.55	25.9	786	0.2	8	
AM	24,532.85	2.63	0.8646	83,230	3,023	19.78	0.72	48.5	1,454	2.1	56	-1.41
BA	19,324.07	26.44	0.7831	104,188	2,436	6.98	0.16	101.3	3,159	1.0	52	
CE	17,178.28	61.70	0.7883	135,945	6,853	14.80	0.75	52.5	2,711	3.2	124	
DF	85,661.32	530.34	*	68,406	871	22.39	0.29	53.4	1,972	0.7	27	
ES	34,493.11	88.21	0.7643	62,236	1,984	15.31	0.49	26	1,440	0.8	37	
GO	28,273,00	20.91	0.7828	35,793	844	5.03	0.12	33.5	1,128	1.4	37	
MA	13,955.68	21.58	0.7257	98,398	2,426	13.83	0.34	66.9	1,873	0.8	38	
MT	39,931.17	3.90	0.7031	27,636	1,029	7.84	0.29	38.7	1,269	1.3	47	
MS	38,925.80	7.87	0.6764	12,969	153	4.62	0.05	24.5	623	0.3	7	
MG	29.223.23	36.30	0.8188	73,813	1,550	3.47	0.07	96.9	3,118	2	63	
PA	18,952.26	6.98	0.7171	124,934	5,274	14.38	0.61	66.7	2,304	4.3	118	-4.3
PB	16,107.61	71.53	0.7788	60,421	1,250	14.96	0.31	34	1,204	0.4	25	
PR	38,772.71	57.79	0.7727	40,797	1,016	3.54	0.09	65.2	1,649	1.6	44	
PE	19,623.66	98.06	0.7878	71,370	5,556	7.42	0.58	29.1	1,198	2.4	86	
PI	15,431.93	13.03	0.7639	32,465	914	9.89	0.28	35.6	1,252	0.5	24	
RJ	44,222.66	396.94	0.8305	129,675	11,406	7.47	0.66	63	2,216	5.6	193	-3.7
RS	40,362.75	40.55	0.6764	38,720	943	3.39	0.08	30.8	1,135	1.5	38	
RN	19,249.73	66.92	0.7887	38,616	1,380	10.93	0.39	36.8	1,140	1.3	39	
RO	25,554.32	7.56	0.6877	26,496	617	14.75	0.34	17.6	597	0.4	13	
RR	23,188.94	2.82	0.7396	21,849	396	34.62.	0.63	22.6	673	0.3	10	
SC	42,149.28	75.76	0.7649	42,026	485	5.79	0.07	58.5	1,718	0.7	18	
SP	48,542.24	186.49	0.8694	366,890	17,702	7.93	0.38	125.9	8,334	4.9	307	
SE	18,442.63	105.76	0.7405	36,046	954	15.54	0.41	36	1,238	0.6	26	
TO	22,932,96	5.73	0.7140	14,939	251	9.39	0.16	21.1	484	0.2	6	

PD: population density; GI: Gini index of gross domestic product of Brazilian states; PC: peak cases; MCA: Maximum case acceleration; PC: peak cases; MDA: Maximum death acceleration; PD: Peak of deaths; DD: deceleration of Deaths; IR: incidence rate; MR: mortality rate; AC: Acre; AL: Alagoas; AP: Amapá; AM: Amazonas; BA: Bahia; CE: Ceará; DF: Distrito Federal; ES: Espírito Santo; GO: Goiás; MA: Maranhão; MT: Mato Grosso; MS: Mato Grosso do Sul; MG: Minas Gerais; PA: Pará; PB: Paraíba; PR: Paraná; PE: Pernambuco; PI: Piauí; RJ: Rio de Janeiro; RN: Rio Grande do Norte; RS: Rio Grande do Sul; RO: Rondônia; RR: Roraima; SC: Santa Catarina; SP: São Paulo; SE: Sergipe; TO: Tocantins. *value not available *in* https://sidra.ibge.gov.br/tabela/5939.

The peak of new cases showed a correlation with PD (r = 0.53, p = 0.005) and GI (r = 0.66, p < 0.001), but no significant correlation was observed with the per capita GDP. The maximum case acceleration correlated with PD (r = 0.41, p = 0.04) and GI (r = 0.61, p = 0.001) (Table 2).

**TABLE 2:** Correlation between socioeconomic variables and variables of the Covid-19 curve in Brazilian states.

*Tabela 1*	GI r	Per capita	PD
	(valor-p)	GDP (reais)	(population / area)
Maximum case acceleration (casos/day^2^)	0.61 (0.001) ^*^	0.16 (0.41) **	0.41 (0.04) *
Peak of cases (N)	0.66 (< 0.001) *	0.19 (0.35) **	0.53 (0.005) *
Maximum death acceleration (deaths/day^2^)	0.56 (0.003) *	0.28 (0.15) **	0.42 (0.03) *
Peak of deaths	0.63 (0.001) *	0.17 (0.40) **	0.49 (0.01) *
Incidence rate (new cases/population *1000)	-0.02 (0.94) *	-0.36 (0.06) **	-0,22 (0,26) *
Mortality rate (new deaths/population*1000)	0.25 (0.22) *	-0.30 (0.13) **	-0.02 (0.91) **

GI: Gini index of gross domestic product of Brazilian states; GDP: Gross domestic product of Brazilian states; PD: Population Density of Brazilian states; *Pearson's correlation; **Spearman correlation.

The highest incidence rates occurred in Amapá, followed by Roraima and Distrito Federal at 36.30, 34.62, and 22.39 cases per 1,000 inhabitants, respectively. The lowest incidence rate was observed in Rio Grande do Sul, 3.39 cases/1,000 inhabitants.

São Paulo had the highest rate of acceleration (125.9 cases/day^2^), and the lowest rate of acceleration was observed in Acre (9.2 cases/day^2^). São Paulo had the highest number of new cases, (8,334 new cases/day), and Acre the lowest, (283 new cases/day).

A total of 14 states were identified during the rising phase of the curve. Piauí, Sergipe, Mato Grosso, Mato Grosso do Sul, Minas Gerais, São Paulo (Figure 2), Santa Catarina, and Rio Grande do Sul are in the first stage of the growth phase, and numbers continue to increase concomitantly with an increase in acceleration. Other states like Paraiba, Tocantins, Ceará, and Pernambuco had a peak of new infections, but the number of cases rose again, surpassing the previous number, and were classified in the ascending phase of the epidemic curve. Goiás and Paraná were in the second stage of the ascending phase.

The states of Acre, Amazonas, Pará, Rondônia, Roraima, Alagoas, Bahia (Figure 2), Maranhão, and Espírito Santo showed stable behavior and were classified in the peak phase of the epidemic curve.

Amapá, Rio Grande do Norte, Distrito Federal, and Rio de Janeiro (Figure 2) presented consistent negative acceleration, classified in the first stage of the deceleration curve; new cases decreased with deceleration; however, they increased without reaching the maximum value. Rio de Janeiro had the highest deceleration value, −71.7 cases/day^2^, day 132 of the series (Figure 2).

### Deaths

**FIGURE 3:**
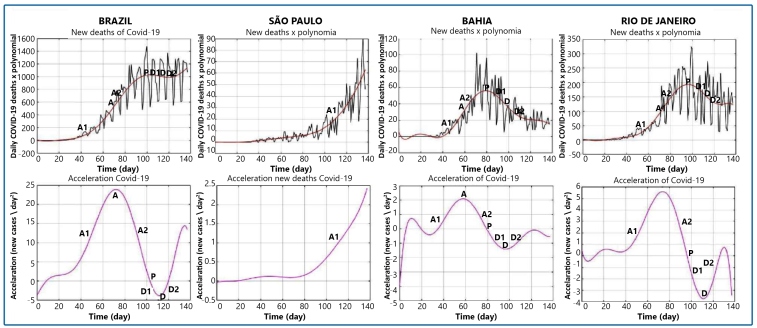
The top figures show the daily deaths by COVID-19 in Brazil, Minas Gerais, Amazonas, and Rio de Janeiro × polynomial. The bottom figures show the daily acceleration of deaths in Brazil, Minas Gerais, Amazonas, and Rio de Janeiro.

On July 11, 2020, the last day of the historical series, Brazil registered 71,469 deaths, with mortality equal to 0.34 deaths/1,000 inhabitants. The country presented a maximum acceleration of 23.9 deaths/day^2^ on May 7th, day 83 of the series. The most recent acceleration, day 132, is equal to 13.4 deaths/day^2^, after a short period of being negative, constituting a peak in plateau (Figure 3). The values by state are listed in Table 1.

A: maximum daily acceleration. A1, first stage of the growth phase; A2: second stage of the growth phase; P: acceleration equal to zero (peak); D1: first stage of the decline phase.

The peak of death was correlated with PD (r = 0.49, p = 0.01) and GI (r = 0.63, p = 0.001). The maximum death acceleration was correlated with PD (r = 0.42, p = 0.03) and GI (r = 0.56, p = 0.003). The per capita GDP did not show any significant correlation with the maximum acceleration of daily deaths or with the maximum value of daily deaths (Table 2).

The incidence rate per 1,000 inhabitants showed a correlation with per capita GDP (rs = −0.36, p = 0.06), without correlation with GI or PD. Mortality rate per 1,000 inhabitants did not correlate with the per capita GDP, GI, or PD (Table 2).

The states of Ceará, Amazonas, and Rio de Janeiro had the highest mortality rate, at 0.75, 0.72, and 0.66 deaths per 1,000 inhabitants, respectively. The lowest mortality rate occurred in Mato Grosso do Sul at 0.05 deaths/1,000 inhabitants (Table 1).

The state of Rio de Janeiro had the highest acceleration rate of deaths (5.6 deaths/day^2^), and Tocantins the lowest acceleration (0.2 deaths/day^2^). São Paulo had the highest number of deaths per day assessed using the polynomial (307 deaths/day). The lowest value occurred in Tocantins at 06 deaths/day.

The states of Rio Grande do Sul, Santa Catarina, Paraná, Minas Gerais (Figure 3), and Mato Grosso are in the first stage of the growth phase of the curve. An increase in deaths accompanied by an increase in daily acceleration was noted. The states of Mato Grosso do Sul, Goiás, Distrito Federal, Paraíba, and Bahia were in the second stage of the growth phase of the death curve, an increase in deaths/day associated with positive acceleration, but with a decrease in the value. São Paulo and Tocantins peaked, but the death curve rose again and, therefore, is in a new phase of acceleration. Ceará was in the descending phase of the curve, but accelerated again.

Sergipe and Roraima showed a peak behavior at the end of the historical series. Alagoas, Pernambuco, Maranhão, and Rondônia showed a peak in plateau.

Rio de Janeiro (Figure 3), Espírito Santo, Rio Grande do Norte, Piauí, Amapá, and Acre were at the end of the first stage of degrowth. Amazonas (Figure 3) and Pará were in the second stage of the descending phase.

## DISCUSSION

Our results demonstrate that the peak of daily cases, acceleration of cases and deaths, and peak of deaths showed a positive correlation with PD and the GI of the GDP of Brazilian states.

PD is mathematically associated with the possibility of greater contact between people and viral transmission^13^ ^,^ ^14^. In addition, social distance is important in flattening the epidemic curve, with a reduction in peak cases and deaths^15^ ^-^ ^17^.

Brazil presented a great acceleration of new cases, and at the end of this series, it was approaching its peak. This curve represents the sum of various epidemics already occurring in the states, in different phases and at different severity levels. Such heterogeneity may be related to the central government’s lack of coordination regarding measures to control the spread of the virus, territorial extent, and the country’s geographical, social, economic, and cultural diversity. In addition, unlike other epidemic diseases, the COVID-19 epidemic in Brazil had its first reports of imported cases (aloctenes), brought by subjects who returned from international trips or foreigners from countries that were themselves in epidemic situations, and later extended to the periphery and smaller cities in the interior of the country^9^.

The results of this study suggest that the worst COVID-19 indicators are related to the PD and GI (inequality indicator), but with no correlation to the per capita GDP, the same behavior observed with social problems more related to inequality than per capita income^18^. Other authors have already described the spatial association between mortality due to COVID-19 and poor living conditions in Northeast Brazil^19^ ^,^ ^20^.

The incidence and mortality rate data should be viewed with caution, as we continue to experience the epidemic, and at this moment, data that reflect the daily variation of newly reported cases and their acceleration are needed, because they reflect the strength or trend of the outbreaks taking daily data into account. Comparing it with previous data is not necessary, as it assesses the absolute slope of the curve. Further, its measurement can be useful in evaluating non-pharmacological measures in response to the COVID-19 pandemic.

Our study did not compare the acceleration rate of case numbers and the reproduction number, a well-established concept in epidemiology, as well as the acceleration rate and moving average variation. This comparison was not the subject of our study.

Polynomial interpolation was used to generate an epidemiological curve, which is usually performed using the moving average. However, in contrast to the moving average, polynomial interpolation allows the calculation of the rate of change and growth rate in cases and deaths^5^. Compared to the growth rate, R is the ratio of cases to infection generation. R is not a rate: there is no timescale involved; the rate of change (growth rate) is a robust measure that describes what is happening at each point of the curve, describing how quickly the number of infections or deaths change daily^21^.

## LIMITATIONS

Our study has the following limitations: First, the design of the ecological study prevented the assessment of causal associations, ecological fallacy, the results of which must be understood as hypotheses. Second, the sample size did not allow the application of a regression model. Last, the characteristics of care for patients or the measures of social distance practiced in each state of the federation were not examined, which may influence the behavior of the epidemiological curve.

## CONCLUSION

Brazilian states presented heterogeneous data curves and were therefore classified at different stages of the epidemic, with socioeconomic inequality and PD being associated with a more rapid exponential growth in new cases and deaths.
